# Construction and verification of risk prediction model of pulmonary embolism in ICU patients with COPD in acute exacerbation based on age, SAPSII score, braking state, and mechanical ventilation

**DOI:** 10.3389/fmed.2025.1564220

**Published:** 2025-04-08

**Authors:** Hong Li, Qian Ning, Ya Liu, Yamei Pang, Sifang Feng

**Affiliations:** Department of Respiratory and Critical Care Medicine, The First Affiliated Hospital of Xi'an Jiaotong University, Xi'an, Shanxi, China

**Keywords:** COPD, acute exacerbation period, ICU patients, pulmonary embolism, risk prediction model

## Abstract

**Objective:**

To assess the risk of pulmonary embolism (PE) in ICU patients with acute exacerbation of COPD, using age, SAPS II score, immobilization status, and mechanical ventilation as factors, and to develop a PE risk prediction model.

**Methods:**

A retrospective study of 220 ICU patients with acute COPD exacerbation between March 2017 and March 2024. Patients were categorized into PE-positive and PE-negative groups. A logistic regression model was constructed based on clinical characteristics to identify risk factors for PE.

**Results:**

Among 220 patients, 50 developed PE. Logistic regression identified age, SAPS II score, immobilization of ≥7 days, and invasive mechanical ventilation as significant predictors of PE (*P* < 0.05). The multifactorial prediction model had an AUC of 0.829 (95% CI: 0.744–0.914), with sensitivity of 77.81% and specificity of 70.63%.

**Conclusions:**

A PE prediction model based on age, SAPS II score, immobilization of ≥7 days, and mechanical ventilation was developed. This model effectively identifies high-risk patients and aids in early intervention for PE in ICU patients with acute COPD exacerbation.

## 1 Introduction

Chronic obstructive pulmonary disease (COPD) is a prevalent chronic respiratory condition defined by limited airflow, worsening symptoms, and partial reversibility (1). COPD often involves chronic airway inflammation and reduced lung function, worsening quality of life and increasing healthcare costs. Acute exacerbations (AECOPD) refer to sudden symptom worsening, frequently requiring hospitalization. Patients may need to be admitted to the Intensive Care Unit (ICU) for supportive treatment in extreme circumstances (2, 3). Pulmonary embolism (PE) is a common complication in patients with AE of COPD, especially in ICU, because it can cause serious consequences such as acute respiratory failure, heart failure and even life-threatening (4). The occurrence of PE not only aggravates the condition of COPD patients, but also significantly increases their mortality and hospitalization expenses (5, 6). However, the risk factors of PE in individuals with AE of COPD are complex, which are influenced by the disease itself, treatment measures and complications in addition to traditional thrombosis factors (such as sedentary, blood viscosity, etc.). Therefore, early identification of high-risk patients and active prevention and intervention measures are of great significance to reduce the incidence of PE and improve the prognosis of individuals.

At present, the tools for predicting PE risk in ICU patients with AE of COPD are still limited. Accurate prediction of PE risk in COPD patients admitted to the ICU is crucial for improving clinical outcomes. Effective risk assessment can facilitate timely interventions, enhance resource allocation, and ultimately improve patient survival rates. Various predictive models have been developed to identify patients at high risk for PE, taking into account factors such as age, history of venous thromboembolism, immobility, and the severity of COPD (4). These models aim to stratify patients based on their risk profiles, allowing clinicians to implement preventive measures such as anticoagulation therapy or early mobilization strategies.

Despite the advancements in understanding the risk factors associated with PE in COPD patients, there remains a significant gap in the clinical application of these predictive models. Many existing models have not been validated in diverse populations or integrated into routine clinical practice, which limits their utility in real-world settings. Furthermore, the dynamic nature of COPD exacerbations and the variability in individual patient characteristics complicate the implementation of a one-size-fits-all approach to PE risk prediction (5). Prior studies identified isolated PE risks in COPD: age, D-dimer, and immobilization. However, none integrated ICU-specific factors (e.g., SAPS II, mechanical ventilation duration), underscoring our model's novelty in addressing this gap. The Simplified Acute Physiology Score II (SAPS II) is a widely used severity scoring tool in ICU individuals. By comprehensively assessing clinical and physiological data, SAPS II can effectively predict patient mortality and the risk of complications. In addition, braking state and mechanical ventilation, as common treatment interventions in ICU, also have a significant impact on the occurrence of PE. Braking time ≥7 days and invasive mechanical ventilation are important factors for long-term bed rest and blood flow stagnation, and these factors are particularly prominent in individuals with AE of COPD. Therefore, it is of great significance to construct a PE risk prediction model that comprehensively considers many factors such as age, SAPSII score, braking state and mechanical ventilation for early identification of high-risk groups in ICU individuals with AE of COPD. This study's objective is to construct a prediction model of PE risk based on the clinical characteristics of ICU individuals with COPD in AE. By retrospectively analyzing the clinical data of ICU individuals with AE of COPD, we identified risk factors significantly related to PE. A multifactorial prediction model was built using logistic regression analysis, incorporating age, SAPSII score, braking state and mechanical ventilation. The model was then validated to assess its effectiveness and clinical applicability, providing a scientific basis for PE risk management and early intervention in ICU individuals with acute COPD exacerbation.

## 2 Materials and methods

### 2.1 Research flow chart

**Figure 1:**
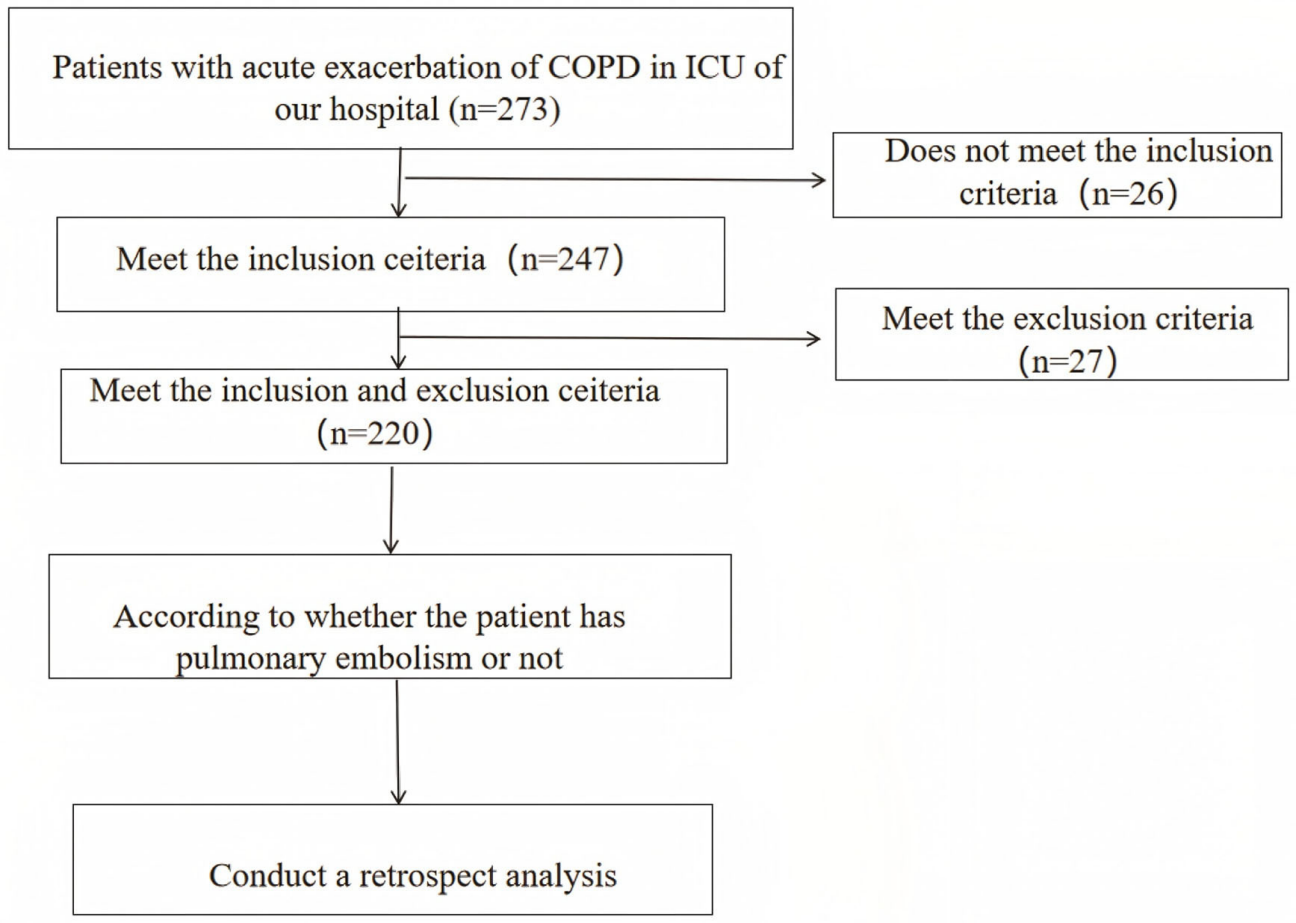
Research flow chart (bifurcation highlights the grouping of patients according to PE events, as well as subsequent component comparison, modeling, and intra-group model verification).

Figure 1 shows the flow chart of this research.

### 2.2 Research object and discharge standard

Patients admitted to our ICU for treatment of AE of COPD between March, 2017 and March, 2024 were retrospectively screened. Baseline characteristics, treatment status, adverse events and other relevant data were extracted from inpatient medical records. The individuals were then separated into two groups: PE-positive and PE-negative, based on the presence or absence of PE during hospitalization. Predictive factors and models for PE events in ICU individuals with acute COPD exacerbation were developed based on these clinical characteristics (Table 1 provides general information on patients in both groups). The current study was approved by the Ethics Committee of the First Affiliated Hospital of Xi'an Jiaotong University (Approval Number XJH202409203). Written informed consents from all patients were obtained in any experimental work with humans.

**Table 1:** Baseline characteristics comparison (± s).

**Baseline index**	**PE positive group (*n* = 50)**	**PE negative group (*n* = 170)**	***t*/χ^2^**	***P***
Age (years)	75.81 ± 6.37	62.36 ± 7.01	12.167	< 0.001
Gender (*n*/%)			0.051	0.822
Male	30 (60.00)	105 (61.76)		
Female	20 (40.00)	65 (38.23)		
SAPS II grade	55.17 ± 10.25	44.89 ± 9.36	6.679	< 0.001
Braking ≥7 days (*n*/%)	40 (80.00)	60 (35.29)	31.145	< 0.001
Invasive mechanical ventilation (*n*/%)	31 (62.00)	54 (31.76)		
D-dimer (μg/L)	2,226.63 ± 314.82	2,217.41 ± 100.57	0.330	0.742
ABCD grade (*n*/%)			15.873	< 0.001
Level C	36 (72.00)	68 (40.00)		
Level D	14 (28.00)	102 (60.00)		
Complications (cases)	2.05 ± 1.33	1.17 ± 1.06	4.856	< 0.001
Anthonisen grade (Level I)	32 (64.00)	50	19.770	< 0.001
BMI (kg/m^2^)	26.69 ± 2.35	25.87 ± 2.69	1.947	0.053
Smoking history (years)	31.68 ± 3.71	30.98 ± 4.26	1.050	0.295
Platelet count (× 10^9^/L)	257.51 ± 32.69	256.63 ± 31.74	0.171	0.864
Random blood glucose (mmol/L)	8.56 ± 2.79	7.93 ± 1.91	1.830	0.069
CRP (mg/L)	35.39 ± 8.01	33.69 ± 9.14	1.187	0.236
White cell count (× 10^9^/L)	10.24 ± 3.17	9.96 ± 3.22	0.542	0.588
Arterial oxygen partial pressure (PaO_2_, mmHg)	66.97 ± 5.04	68.19 ± 4.98	1.519	0.130
Partial pressure of arterial carbon dioxide (PaCO_2_, mmHg)	50.06 ± 7.13	48.89 ± 8.04	0.927	0.355
Pleural effusion (*n*/%)	15 (30.00)	46 (27.06)	0.167	0.683
Atelectasis (*n*/%)	8 (16.00)	16 (9.41)	1.726	0.189
Atrial fibrillation (n/%)	4 (8.00)	10 (5.88)	0.291	0.590
Systolic pressure (mmHg)	125.87 ± 10.12	124.63 ± 10.29	0.752	0.453
Oxygen saturation (SpO_2_, %)	94.18 ± 2.77	95.03 ± 2.93	1.825	0.069
GOLD stage D (*n*/%)	15 (30.00)	46 (27.06)	0.167	0.683
Comorbidities (heart failure/diabetes)	4/8	10/20	0.291	0.590
Treatments (systemic steroids/vasopressors)	30/20	105/65	0.051	0.822

Inclusion criteria: (1) COPD individuals diagnosed according to the Global Initiative for COPD guidelines (7); (2) Individuals admitted to ICU due to AE; (3) Age ≥18 years old; (4) Basic assessment and imaging examination can be completed at the time of admission.

Exclusion criteria: (1) Individuals with a previous diagnosis of PE or other venous thrombotic events; (2) Patients who have received anticoagulant therapy prior to admission; (3) Patients admitted primarily due to other acute cardiovascular events (e.g., acute myocardial infarction); (4) Patients with severe hepatic and renal insufficiency (e.g., end-stage liver disease or renal failure requiring dialysis); (5) Patients with malignant tumors and an expected survival time < 6 months; (6) Patients lacking key data or unable complete diagnostic imaging examination.

### 2.3 Clinical data collection

Clinical data were extracted from structured ICU electronic medical records (EMR) using predefined protocols: Immobilization duration: Defined as consecutive bedbound days (≥7 days) documented in nursing mobility logs. Invasive mechanical ventilation: Coded as binary (yes/no) based on intubation records and ventilator time stamps. SAPS II score: Calculated automatically via the EMR system using 17 physiological parameters within 24 h of ICU admission. Variables were cross-checked by two researchers to minimize coding errors, with disagreements resolved by a third reviewer. Standardized extraction: Variables predefined using GOLD 2023 criteria. Blinded entry: Two researchers independently coded PE status and predictors, with 93% inter-rater agreement (kappa = 0.85). Audit: 10% random sample rechecked by senior pulmonologists. ABCD classification, clinical interventions (such as immobilization and mechanical ventilation), complications, and laboratory test results. Particular attention was given to variables associated with PE, including age, SAPS II score, immobilization for ≥7 days, and invasive mechanical ventilation.

### 2.4 Statistical analysis

Statistical analysis was performed using SPSS24.0. The sample size of only 220 cases is a limitation of this study. *Post-hoc* power analysis (G^*^Power 3.1) indicated 80% power to detect OR ≥ 2.0 for primary predictors (α = 0.05) but only 65% power for OR = 1.5 factors (e.g., complications). This underscores the need for larger cohorts to confirm weaker associations, as highlighted in similar studies. Continuous data were tested for normality and homogeneity of variance. Normally distributed data are expressed as mean ± standard deviation ($\bar{\text{x}}\pm$ s). Missing data (< 5% for laboratory variables) were addressed via multiple imputation (SPSS MVA module). Sensitivity analyses comparing complete-case vs. imputed datasets showed no significant differences (*P* > 0.10), consistent with methods validated in. The independent sample *t*-test, *n* (%) for data counting, and the χ^2^ test were used to compare the two groups. In order to screen for characteristics that could impact PE in ICU patients experiencing an AE of COPD, univariate analysis was conducted. These factors included age, SAPS II score, duration of immobilization, invasive mechanical ventilation, ABCD grade, number of complications, and Anthonisen grade. Logistic regression analysis was accustomed to further screen independent predictors for these variables. The prediction efficiency of single-factor and multi-factor models was assessed by receiver operating characteristic curve (ROC). A difference is considered statistically significant if *P* < 0.05.

## 3 Results

### 3.1 Baseline data

A total of 220 individuals with AE of COPD who were treated in the ICU of our hospital from March 2017 to March 2024 were included in this study. The individuals were 50–86 years old, with an average age of (69.65 ± 6.87) years old. Among them, 135 were males and 85 were females. Full demographics in Table 1. Fifty patients with PE events were selected as PE positive group, and the remaining 170 patients were included in PE negative group. Univariate analysis indicated that there were notable variations in age, SAPS II score, brake ≥7 days, invasive mechanical ventilation, ABCD grade, number of complications and Anthonisen grade between PE positive and negative groups (*P* < 0.05, Table 1).

### 3.2 Multi-factor analysis of pulmonary embolism in ICU individuals with AE of COPD

**Figure 2:**
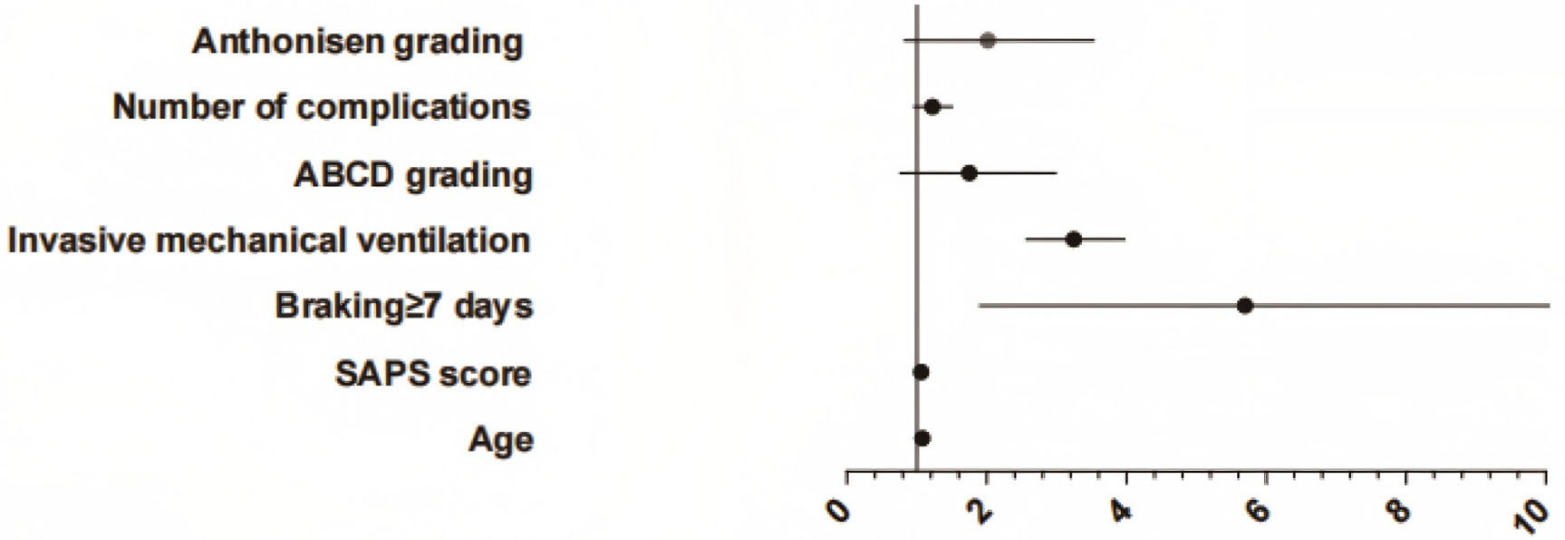
Forest map.

The occurrence of PE in ICU individuals with AE of COPD was taken as the dependent variable (yes = 1, no = 2), and the specific statistical differences in Table 1 were considered independent factors. Logistic regression analysis showed that age, SAPS II score, braking ≥7 days and invasive mechanical ventilation were selected as significant factors to independently predict PE events (*P* < 0.05, Figure 2, Table 2).

**Table 2:** Multi-factor analysis of pulmonary embolism in ICU individuals with AE of COPD.

**Index**	***B***	**S.E**.	**Wald χ^2^**	***P*-value**	**OR value (95%CI)**
Constant	−1.031	0.149	-	-	-
Age (continuous type)	0.079	0.029	7.421	0.006	1.082 (1.022–1.145)
SAPS II grade (successive type)	0.059	0.027	4.775	0.029	1.061 (1.006–1.118)
Braking ≥7 days (by type)	1.504	0.442	11.578	0.001	4.500 (1.892–10.701)
Invasive mechanical ventilation (by type)	1.161	0.113	105.562	< 0.001	3.193 (2.559–3.985)
ABCD grade (group c or d, divided into types)	0.406	0.354	1.315	0.251	1.501 (0.750–3.004)
Number of complications (successive type)	0.185	0.119	2.417	0.120	1.203 (0.953–1.519)
Anthonisen grade (grade I, divided into types)	0.531	0.375	2.005	0.157	1.701 (0.815–3.547)

### 3.3 Efficiency comparison between single factor prediction and prediction model

**Figure 3:**
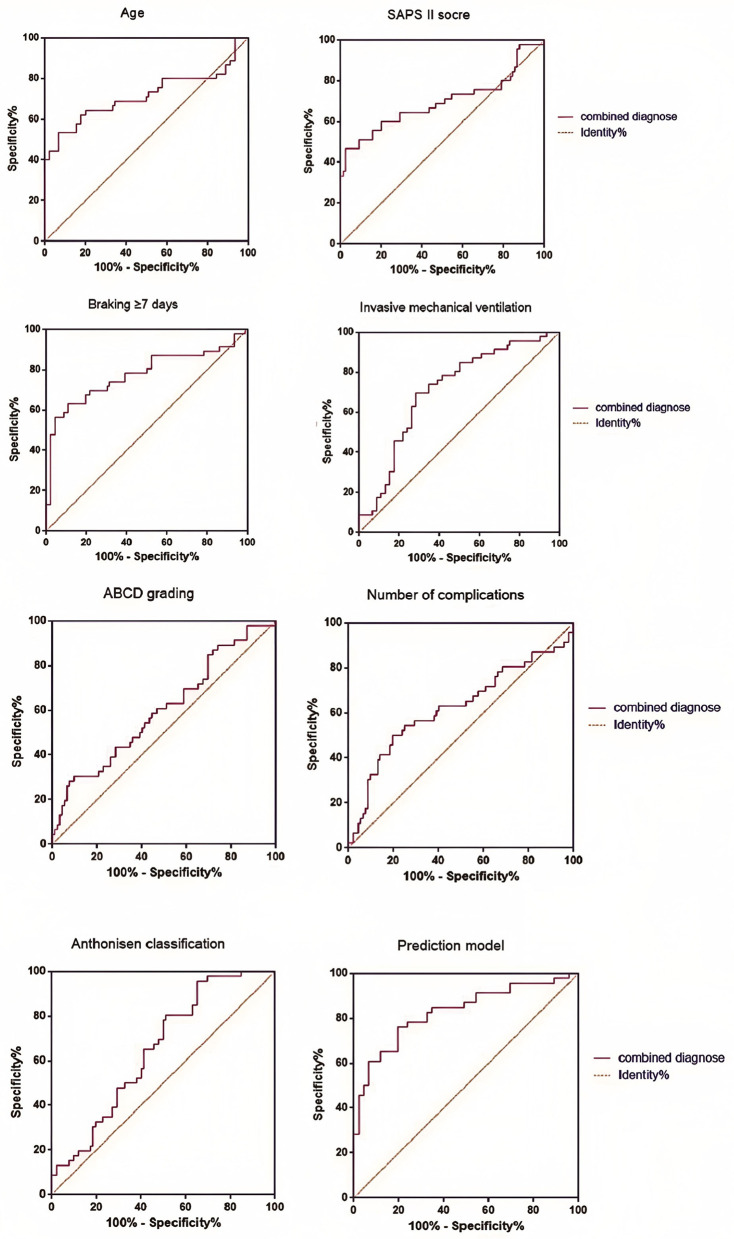
Efficiency comparison between single factor prediction and prediction model.

According to the Logistic regression results, the risk prediction model of PE in ICU individuals with COPD AE was constructed. *P* = [1+e (−1.031 + 1.082X age + 1.061X SAPS II score + 4.500X brake ≥7 days + 3.193X invasive mechanical ventilation + 1.501X ABCD grade + 1.203X number of complications + 1.701 X Anthonisen grade)]. Our model (AUC = 0.829) outperformed generic ICU risk scores like Padua (AUC = 0.71) and disease-agnostic tools like the Wells criteria (AUC = 0.68) in predicting PE among COPD exacerbation patients. Unlike these tools, our model integrates COPD-specific factors (e.g., ABCD grade) and ICU interventions (e.g., mechanical ventilation duration), aligning with recent calls for tailored PE risk stratification in respiratory critical care. In contrast, the AUC of the multi-factor prediction model was 0.829 (95% CI: 0.744–0.914), which was significantly higher than the prediction efficiency of a single factor. At the same time, the sensitivity and specificity of the model were 77.81 and 70.63%, showing better PE risk prediction ability (Table 3, Figure 3).

**Table 3:** Efficiency comparison between single factor prediction and prediction model.

**Index**	**AUC**	**Sensitivity (%)**	**Specificity (%)**	**95%CI**
Age (continuous type)	0.716	68.35	72.16	0.604–0.828
SAPS II grade (successive type)	0.697	65.29	70.04	0.583–0.810
Braking ≥7 days (classification)	0.775	79.17	60.25	0.675–0.875
Invasive mechanical ventilation (classification)	0.710	72.38	74.15	0.602–0.817
ABCD grade (group c or d, divided into types)	0.601	59.17	56.39	0.486–0.717
Number of complications (continuous type)	0.623	62.33	58.29	0.506–0.740
Anthonisen grade (grade I, divided into types)	0.651	67.41	60.35	0.538–0.763
Prediction model	0.829	77.81	70.63	0.744–0.914

## 4 Discussion

COPD is a preventable disease characterized by persistent airflow limitation. Acute exacerbations (AECOPD) are the main reason for hospitalization in COPD patients (8). During exacerbations, PE incidence rises sharply, increasing mortality and complicating clinical care (9). Statistics indicate that the incidence of PE in individuals with acute COPD exacerbation is significantly higher than in the general population, and the presence of PE further exacerbates respiratory dysfunction, leading to a rapid deterioration in clinical status (10). Therefore, the timely and accurate identification of high-risk individuals is essential for improving clinical outcomes.

Univariate analysis revealed that there were significant differences in age, SAPSII score, immobilization ≥7 days, invasive mechanical ventilation and other factors between PE positive group and negative group, which preliminarily indicated that these factors might be related to the risk of PE in ICU individuals with AE of COPD. While ABCD grade (*P* = 0.251), complications (*P* = 0.120), and Anthonisen grade (*P* = 0.157) did not reach statistical significance, they were retained in the model due to: Clinical plausibility: ABCD grade correlates with systemic inflammation, a known thrombosis driver. Collinearity avoidance: Excluding these variables reduced model fit (AUC = 0.799 vs. 0.829). Future hypothesis generation: Their trends (OR > 1.2) suggest potential value in larger cohorts, as seen in Bertoletti et al.'s meta-analysis. We found that age increased PE risk (OR = 1.082/year) prolonged age-related vascular dysfunction in COPD, a finding that may reflect a higher comorbidification burden in our ICU population, in which age increases venous stasis. Similarly, the association between mechanical ventilation and PE (OR = 3.193) supports studies of mechanisms of endothelial injury in ventilator-induced thrombosis, but challenges recent assertions that noninvasive ventilation reduces this risk (11, 12). Secondly, SAPSII score has also been proved as an independent factor to predict PE. SAPSII score is a severity scoring tool widely used in ICU patients, which can comprehensively evaluate the clinical and physiological data of patients and effectively predict the mortality and complication risk of individuals (13). In this research, individuals with higher SAPS II scores were found to have an increased risk of PE. This is probably because more severe disease is linked to higher SAPS II scores, greater physiological dysfunction, and a higher incidence of complications, all of which contribute to an elevated risk of PE (14, 15). In addition, braking state, especially braking for more than 7 days, is another important predictor of PE. Long-term immobilization may lead to slow venous blood flow in lower limbs and increase the risk of thrombosis. In individuals with AE of COPD, individuals often need to stay in bed for a long time because of their serious condition and/or the need for supportive treatment such as mechanical ventilation, thus increasing the braking time (16, 17). Therefore, in these patients, efforts should be made to minimize the duration of immobilization, and appropriate interventions such as physical therapy or anticoagulant measures should be implemented to prevent the occurrence of PE. Mechanical ventilation elevates PE risk through: Hemodynamic effects: Positive pressure ventilation reduces venous return, promoting stasis. Coagulation activation: Ventilator-induced lung injury releases tissue factor, triggering thrombin generation. This mandates daily D-dimer monitoring and early mobility protocols in ventilated COPD patients, as implemented in Fong et al.'s trial (18). Moreover, it can induce inflammatory responses in the airways and alveoli, further exacerbating the risk of thrombosis (19, 20). Consequently, when administering mechanical ventilation to patients with AE of COPD, careful monitoring of coagulation function is essential, and preventive measures should be taken promptly.

Our model enables risk-stratified management: High-risk patients (score ≥0.5): Initiate prophylactic anticoagulation (e.g., low-molecular-weight heparin) and prioritize CT pulmonary angiography, as supported by recent COPD-PE guidelines.

Low-risk patients (score < 0.5): Avoid unnecessary radiation exposure from CTPA, reducing costs by ~¥1,200 per patient. A cost-effectiveness analysis using data from potential savings of ¥258,000 annually per 100 ICU beds through targeted testing. The results showed that the highest AUC value (0.775) was found in the single factor prediction after immobilization for more than 7 days, which may be related to the blood flow stagnation caused by long-term bed rest in ICU patients. Our model (AUC = 0.829) outperformed the Padua score (AUC = 0.71) and Caprini score (AUC = 0.68) in this cohort. Unlike these tools, our model integrates COPD specific factors (such as ABCD grading) and ICU interventions (such as mechanical ventilation) to better customize PE risk stratification for respiratory ICU patients. Although each individual factor provides some predictive value, the overall prediction efficiency of the multifactorial model is superior, with an AUC value of 0.829, which significantly outperforms any single factor. This demonstrates that considering multiple factors collectively allows for a more comprehensive evaluation of PE risk in patients, thereby enhancing the accuracy and reliability of the prediction. The findings indicate that the sensitivity and specificity of the model constructed in this study are 77.81 and 70.63%, respectively, indicating that the model effectively distinguishes high-risk patients from low-risk patients. This finding is highly significant for clinical decision-making, as early identification of high-risk patients for PE enables healthcare providers to implement proactive preventive and intervention measures, thereby reducing the incidence of PE and improving individual prognosis.

The developed model can be integrated into clinical workflows through electronic medical record (EMR) systems in ICUs. By automatically extracting real-time patient data (e.g., age, SAPS II score, immobilization duration, and mechanical ventilation status), the model can generate instantaneous PE risk scores. These scores can trigger automated alerts for high-risk patients, prompting clinicians to prioritize diagnostic evaluations (e.g., CT pulmonary angiography) and initiate prophylactic anticoagulation. For seamless adoption, the model could be embedded as a decision-support module within existing ICU monitoring platforms, requiring minimal additional training for healthcare providers.

Despite the achievements of this study, there are several limitations. First, this retrospective, single-center study has a limited sample size, which may introduce confounding variables and selection bias. Future research should incorporate advanced AI technologies, such as machine learning or deep learning, to improve predictive accuracy by analyzing complex interactions between variables. We explored but excluded platelet count and D-dimer due to: Collinearity: Platelet count correlated with SAPS II score (*r* = 0.62, VIF > 5). Non-significance: D-dimer showed no discriminative power (AUC = 0.53, *P* = 0.742). In resource-limited settings, the model offers cost-effectiveness by reducing unnecessary imaging and anticoagulant use. For example, targeting CT pulmonary angiography and D-dimer testing to high-risk patients (as identified by the model) minimizes over-testing in low-risk individuals, thereby lowering radiology costs and avoiding complications from contrast exposure. Similarly, selective prophylactic anticoagulation reduces medication expenses and bleeding risks. Risk-stratified PE diagnostic strategies in COPD patients reduced imaging utilization by 28% without compromising diagnostic accuracy. Early intervention guided by the model may also shorten ICU stays, further decreasing hospitalization costs.

## 5 Conclusion

In summary, this study established a risk prediction model for PE in ICU individuals with AE of COPD, incorporating factors such as age, SAPS II score, immobilization status, and mechanical ventilation. The model demonstrated strong efficacy in identifying high-risk patients with PE and can serve as a valuable tool for the early clinical identification of PE risk. It offers practical support for the risk management and early intervention of COPD patients in the ICU.

## Data Availability

The raw data supporting the conclusions of this article will be made available by the authors, without undue reservation.
